# Effect of virtual reality exposure therapy on social participation in people with a psychotic disorder (VRETp): study protocol for a randomized controlled trial

**DOI:** 10.1186/s13063-015-1140-0

**Published:** 2016-01-13

**Authors:** Roos Pot-Kolder, Wim Veling, Chris Geraets, Mark van der Gaag

**Affiliations:** Parnassia Psychiatric Institute, Zoutkeetsingel 40, 2512 HN Den Haag, The Netherlands; Department of Clinical Psychology, VU University and EMGO Institute for Health and Care Research, Van der Boechorststraat 1, 1081 BT Amsterdam, The Netherlands; University of Groningen, UMC Groningen, University Center of Psychiatry, Hanzeplein 1, 9700 RB Groningen, The Netherlands

**Keywords:** Psychosis, Schizophrenia, Paranoia, Interaction anxiety, Virtual Reality, Exposure, Social participation, Therapy

## Abstract

**Background:**

Many patients with a psychotic disorder participate poorly in society. When psychotic disorders are in partial remission, feelings of paranoia, delusions of reference, social anxiety and self-stigmatization often remain at diminished severity and may lead to avoidance of places and people. Virtual reality exposure therapy (VRET) is an evidence-based treatment for several anxiety disorders. For patients with a psychotic disorder, the VRETp was developed to help them experience exposure to feared social situations. The present study aims to investigate the effects of VRETp on social participation in real life among patients with a psychotic disorder.

**Methods/design:**

The study is a single-blind randomized controlled trial with two conditions: the active condition, in which participants receive the virtual reality treatment together with treatment as usual (TAU), and the waiting list condition, in which participants receive TAU only. The two groups are compared at baseline, at 3 months posttreatment and at 6 months follow-up. All participants on the waiting list are also offered the virtual reality treatment after the follow-up measurements are completed. The primary outcome is social participation. Secondary outcomes are quality of life, interaction anxiety, depression and social functioning in general. Moderator and mediator analyses are conducted with stigma, cognitive schemata, cognitive biases, medication adherence, simulator sickness and presence in virtual reality. If effective, a cost-effectiveness analysis will be conducted.

**Discussion:**

Results from the posttreatment measurement can be considered strong empirical indicators of the effectiveness of VRETp. The 6-month follow-up data may provide reliable documentation of the long-term effects of the treatment on the outcome variables. Data from pre-treatment and mid-treatment can be used to reveal possible pathways of change.

**Trial registration:**

Current Controlled Trials: ISRCTN12929657. Date of registration: 8 September 2015

## Background

A large number of patients with a psychotic disorder participate poorly in society, even if their psychotic symptoms have been treated successfully. Unemployment is high at 80–85 % [1] and about 75 % do not have a relationship with a partner [2]. A study comparing the social network of young people with early psychosis and matched controls showed that the psychosis group had smaller networks, fewer friends, fewer people to turn to in a crisis, and a greater likelihood of service providers as members [3]. Social network size is also associated with the likelihood of in-patient treatment and with the number of services used by psychotic patients [4]. Social isolation hinders patients in multiple areas of functioning, such as developing and maintaining a social network, the ability to function in work-related environments, and even in performing daily tasks needed for independent living (e.g., shopping for groceries). When psychotic disorders are in partial remission, the remaining feelings of paranoia and delusions of reference often cause patients to avoid places and people. Moreover, this conditioned avoidance does not improve with antipsychotic medication [5].

**Table 1:** Measurement objectives

Outcome	Measurement	T0	T1	T2	T3	T6
	Interview (i) Self-report (s)	Baseline	Session 4	Session 8	Posttreatment	Follow-up
Social participation	Experience Sampling Method (s)	x			x	x
Paranoia	GPTS (s)	x	x	x	x	x
Interaction anxiety	SIAS (s)	x	x	x	x	x
Depression	BDI-II (s)	x			x	x
Stigma	ISMI (s)	x			x	x
Schemas	BCSS (s)	x			x	x
Safety behavior	SBQ (i)	x			x	x
Cognitive biases	DACOBS (s)	x			x	x
Social functioning	SOFAS (i)	x			x	x
Quality of life	MANSA (i)	x			x	x
Cost-effectiveness	TIC-P (i)	x			x	x
Cyber sickness	SSQ (s)		x	x		
Presence	IPQ (s)		x	x		
Medication adherence	BARS (i)	x	x	x	x	x

*GPTS* Green et al. Paranoid Thought Scales, *SIAS* Social Interaction Anxiety Scale, *BDI-II* Beck Depression Inventory, *ISMI* Internalized Stigma of Mental Illness, *BCSS* Brief Core Schema Scales, *SBQ* Safety Behavior Questionnaire – persecutory delusions, *DACOBS* Davos Assessment of Cognitive Biases Scale, *SOFAS* Social and Occupational Functioning Assessment Scale, *MANSA* Manchester Short Assessment of quality of life, *TiC-P* Trimbos/iMTA questionnaire for Costs associated with Psychiatric illness, *SSQ* Simulator Sickness Questionnaire, *IPQ* Igroup Presence Questionnaire, *BARS* Brief Adherence Rating Scale

### Exposure

The evidence-based psychological treatment for experiencing fear and paranoia in social situations is cognitive behavioral therapy (CBT) with exposure in vivo, such as the therapist taking a patient shopping or exposing the patient to public transportation. This form of treatment in vivo has several limitations. First, the social world in which in vivo exposure takes place cannot be experimentally manipulated. Second, exposure therapy in vivo is costly and not readily available in most mental healthcare institutes. Third, therapy sessions are used to prepare exposure exercises, which the patient is expected to perform between sessions; however, even with careful tailoring to the patients’ capabilities, it is often difficult for patients to do these exercises as planned. Fourth, not all patients tolerate exposure in vivo.

### Anxiety disorders and VRET

Virtual reality exposure therapy (VRET) is an evidence-based treatment for several anxiety disorders [6]. It has the potential to be an affordable and accessible form of treatment to enhance social participation and wellbeing for patients suffering from a psychotic disorder and social withdrawal. In virtual worlds fear is experienced similar to the in vivo experience. It is the experience of being there (known as ‘presence’), which the three-dimensional virtual reality (VR) environment creates, together with a narrative about the environment, that enables people to feel and behave as they would in real life. This principle makes it possible to overcome fear and practice new behavior in a virtual environment [7]. An advantage of VR is that people find it easier to start exposure, since they know there is no real threat to their safety [8]. In students suffering from fear of spiders, 81–89 % chose VR exposure over in vivo exposure [9]. A study comparing VR exposure vs. in vivo exposure in specific phobias showed that 76 % of the patients chose VR exposure over in vivo exposure [10]. The refusal rate for in vivo exposure (27 %) was higher than that for the VRET (3 %). In total, 90.4 % of the patients that preferred VR exposure said they did so because they were too afraid to confront the real situation or to object. These results suggest that the availability of VR exposure may increase the number of patients who are willing to engage in exposure-based therapy.

### VRET in psychosis

In a VRET treatment protocol, patients are gradually exposed to controlled social environments that induce fear and in which individually tailored exposure exercises can be designed. The ability to provide fear-inducing VR social environments is partially dependent on the availability of anxiety-provoking stimuli in the software of the virtual worlds. The present study will assess whether the currently used anxiety-provoking stimuli sufficiently match the stimuli asked for by patients during treatment.

An experimental virtual world was developed. The ecological validity of the VR environment has been demonstrated. For patients suffering from psychosis a significant correlation was found between paranoia and social interaction anxiety in real life and paranoid thoughts about the avatars in the VR world [11]. A higher degree of paranoia was found when more avatars were present, when avatars had hostile facial expressions, and when more of the avatars had a different ethnicity [12].

Preliminary findings using VRET with psychosis show that patients experiencing paranoia are willing to participate in VR environments, that they report paranoid thoughts about the virtual people (avatars), but at the same time are willing to confront the fearful situation [11, 13]. Social virtual environments have the potential to enhance CBT by helping patients recovering from psychosis to understand the role of avoidance and safety behaviors in the maintenance of interaction anxiety and paranoia. Additionally, it can enhance their confidence to carry out real-life behavioral experiments [14].

### Side effects and safety

A phenomenon called simulator sickness (also known as cyber sickness) can occur when using VR applications. Symptoms are similar to those of motion-induced sickness, but tend to be less severe and have a lower incidence [15]. It is suggested that simulator sickness sensations can be at least partially explained by an overlap with anxiety symptoms [16]. A pilot study on virtual reality and psychosis showed low symptoms of simulator sickness, and no significant increase between pre- and post-measurement [11]. Since simulator sickness is known to increase with the duration of exposure [15], measurement of these symptoms will be included in the study protocol.

No adverse effects were found in studies using VR to expose psychotic patients to virtual social environments. A study with 20 psychotic patients diagnosed with first-episode psychosis experiencing at least moderate paranoia found that VR did not lead to more anxiety or physical complaints directly after the experiments; follow-up 1 week later showed that no patients reported an increase in intrusive negative thoughts, unpleasant emotions or behavioral changes as a consequence of the VR experience [16]. Similar results were found in a study of 21 patients with at-risk mental state for psychosis [8]. Our own pilot study confirmed that patients did not become more psychotic as a result of exposure to our virtual social environment [11].

### Objectives

As this is a new form of treatment for social withdrawal in psychosis, the first step is to demonstrate the effect of VRET compared to a waiting list condition on social participation in real life. Objective social participation is defined as the time spent in social situations with other people and the time spent interacting with other people in everyday life. Subjective social participation is how patients experience these social situations; this experience is expressed as momentary paranoia, perceived social threat and event stress, as experienced in situations with other people.

We hypothesize that, after applying the intervention for patients with paranoia (VRETp), they will show improved social participation.

### Primary objective

To determine the effectiveness of VRETp in patients with psychosis, defined as improved social participation.

Hypothesis 1: The amount of time spent with other people will increase, as measured 60 times a week in real life.

Hypothesis 2: Momentary paranoia, perceived social threat and event stress as experienced in social situations will decline, as measured 60 times a week in real life.

### Secondary objectives

Secondary objectives are to investigate:

1. the acceptability of VRETp for patients and therapists
2. the effects on interaction anxiety, depression, quality of life and social functioning in general
3. the moderating and mediating effects of stigma, schemata about the self and others, cognitive biases, medication adherence, simulator sickness and experienced presence in the VR environment
4. the cost-effectiveness of VRETp

## Methods

### Participants

Included are patients diagnosed with a psychotic disorder at seven mental health institutions in the Netherlands: a list of study sites can be obtained from the corresponding author. Written informed consent is obtained from each participant.

### Inclusion criteria

To be eligible to participate, patients must meet all of the following criteria:

- DSM IV diagnosis of a psychotic disorder according to the Mini International Neuropsychiatric Interview (MINI)
- Avoiding either shops, streets, public transportation or bars/restaurants as assessed by the Safety Behavior Questionnaire (SBQ)
- A paranoia score (>40) as assessed by the Green et al. Paranoid Thoughts Scale (GPTS)
- Age 18–65 years

### Exclusion criteria

- IQ ≤70. IQ must be established by a valid instrument, such as the Wechsler Adult Intelligence Scale or Wechsler Intelligence Scale for Children. Information on IQ can be found in the status chart of the patient. In case of doubt, the short form of the WAIS III is used to assess IQ
- Insufficient command of the Dutch language
- Epilepsy. If no epilepsy is mentioned in the patient status, this is checked with the patient

### Measurement instruments

#### Social participation (primary outcome)

Social participation is measured with the PsyMate experience sampling device. This form of Experience Sampling Measurement (ESM) has high ecological validity [17]. ESM is a self-assessment technique using random time sampling, and has the advantage that it can assess mental state and social context in everyday life as it occurs. Because the ESM assesses ‘at the moment’ it is less vulnerable for recall bias and a valuable instrument to assess clinical phenomena in the real world [18]. ESM is effective for patients with a psychotic disorder with current symptoms, as well as for patients with a psychotic disorder in remission [19]. A review of studies using the ESM shows it to be valid for measuring situational characteristics, such as social environment [20]. Event stress, social environment and company (time spent in company with others and the kind of company) are operationalized in accordance with the work of Collip et al. [19]. Research on the (social) context of delusions in schizophrenia shows the ESM to be a valid aid in collecting data of daily life social situations [21]. Momentary paranoia, using four established ESM items, is a valid state measure to assess paranoia [22]. Perceived social threat, using four established ESM items, is a valid measure to assess perceived social threat as a more subtle indicator of paranoia [19]. An overview of measurement objectives can be found in Table1.

### Eligibility measurements

#### Psychotic disorders

The MINI-Plus interview is used for diagnosing lifetime psychotic disorders. This interview provides a reliable DSM diagnosis for psychotic disorders. The good psychometric characteristics of the MINI (-Plus) make it a good choice for research purposes [23–25].

### Paranoid thoughts

Paranoia symptoms are assessed with the GPTS [26]. The GPTS consists of 32 items divided into two 16-item scales, assessing ideas of social reference and persecution. Good internal consistency and validity are established for both of the scales and their dimensions [26].

### Safety behavior

The Safety Behavior Questionnaire – persecutory delusions (SBQ) is used to assess safety behaviors (such as avoidance) for social situations [27]. An action was considered to be safety behavior if the patient reported that it had been carried out with the aim to reduce a persecutory threat. The patient was asked to rate the frequency of the safety behavior over the last month on a 4-point scale. Psychometric properties of the SBQ range from poor to excellent [27].

### Secondary outcome measures

#### Quality of life

The Manchester Short Assessment of quality of life (MANSA) is a brief instrument used to assess quality of life, focusing on satisfaction with life as a whole and with life domains. Its psychometric properties are satisfactory [28].

### Interaction anxiety

Interaction anxiety symptoms were assessed with the Social Interaction Anxiety Scale (SIAS) [29]. The SIAS consists of 19 items that assess the tendency to fear and avoid social situations. Responses can range from 0 (not at all) to 4 (extremely). The SIAS has good psychometric properties [29, 30].

### Social functioning

The Social and Occupational Functioning Assessment Scale (SOFAS) is used to subjectively assess and rate social and occupational functioning, but not psychological symptoms [31, 32].

### Depression

The Beck Depression Inventory-II (BDI-II) consists of 21 items assessing symptoms and level of depression over the past 2 weeks. The BDI-II is a psychometrically sound instrument [33].

### Moderators and mediators

#### Stigma

The Internalized Stigma of Mental Illness (ISMI) is a 29-item questionnaire that measures self-stigma among persons with psychiatric disorders. The ISMI shows reliability and validity [34].

### Schemata

Schemata of self and others (Brief Core Schema Scales; BCSS) have 24 items concerning beliefs about the self and others that are assessed on a 5-point rating scale (0–4). The BCSS has good psychometric properties [35].

### Cognitive biases

The Davos Assessment of Cognitive Biases Scale (DACOBS) has seven independent subscales each with six items; jumping to conclusions, belief inflexibility bias, attention for threat bias, external attribution bias, social cognition problems, subjective cognitive problems and safety behavior. The DACOBS is reliable and valid for use in clinical practice and research [36].

### Medication adherence

The Brief Adherence Rating Scale (BARS) is an instrument used to assess the antipsychotic medication adherence of patients with a psychotic disorder. The BARS consists of four items: three questions and an overall visual analog rating scale to assess the proportion of doses taken by the patient in the past month (0–100 %). The psychometric properties are adequate [37].

Participants and their psychiatrists are asked not to change any medication during the trial.

### Simulator sickness

The Simulator Sickness Questionnaire (SSQ) was developed to accommodate symptoms specific to simulator technology. The SSQ requires the user to report the subjective severity of symptoms such as general discomfort, headache, eyestrain and fatigue [15]. Although its psychometric properties are adequate, there is room for improvement [38].

### Presence

The Igroup Presence Questionnaire (IPQ) consists of 14 items assessing sense of presence in virtual environments. Responses are made on a 7-point Likert scale. The IPQ has demonstrated good psychometric properties [39].

### Cost-effectiveness

The Trimbos/iMTA questionnaire for Costs associated with Psychiatric illness (the TiC-P) is designed for self-report by patients with a mental disorder. The questionnaire focuses on establishing direct medical costs and productivity costs during paid or unpaid work. The psychometric properties are reported to be adequate [40].

## Design

The study is a single blind randomized controlled trial (RCT) with two conditions: (i) the active condition in which subjects receive the VR treatment besides treatments as usual (TAU) and (ii) the waiting list condition during which subjects receive TAU only. All participants on the waiting list are also offered the VR treatment after follow-up measurements have been completed. The two groups are compared at baseline, at 3 months posttreatment and at 6 months follow-up. The waiting list condition receives the VRET treatment after 6 months.

### Power and sample size calculation

In this RCT, the effect of VRETp on social participation is investigated by comparing this treatment with a waiting list condition. Social participation is assessed with the PsyMate experience sampling method (ESM; see “Measurements”). Self-assessments are rated on a 7-point Likert scale. Main outcome items are social environment and company, perceived social threat in company, event stress, and momentary paranoia. Estimated mean scores and standard deviations (SD) are not readily available because there are no previous VRETp studies with psychotic patients.

Using an estimated (moderate) effect size of 0.5 with a power of 0.8, an alpha of 0.05 and a two-sided independent *t* test, yields *n* = 64 in each group. Therefore, at least 64 participants need to be included in each arm (total *n* = 128 patients). As attrition is estimated at about 20 %, 80 participants need to be included in each condition (total *n* = 160 patients).

### Procedure

Patients with a chart diagnosis of schizophrenia, schizophreniform disorder, schizoaffective disorder, delusional disorder or psychotic disorder not otherwise specified (NOS), will be informed about the study by their treating specialist and asked to participate. Each patient is asked for written permission to be contacted by the researchers if eligible for VRETp. If eligible, the treating specialist discusses participation of the patient in the study with the remainder of the team and gives permission to the researchers to contact the patient. When permission is given, additional information is provided and patients have 2 weeks to consider whether they really wish to participate. The VRETp treatment is additional to current treatment and declining to participate has no negative consequences at all for patients. For each of the participating organizations an independent specialist is available for patients to contact. Contact information of this specialist is available in the information letter given to the patients of the participating organizations.

Informed consent from all participants is obtained before assessment by the researcher. The MINI-Plus interview is used to diagnose psychotic disorders. Patients who are willing to participate are screened for avoidance behavior (SBQ) and for paranoia using the GPTS (cutoff >40) of Green et al. Baseline measurements are obtained. Randomization is used to allocate a patient to either the VRETP or to the waiting list condition. Patients allocated to the VRETp group start their treatment, which consists of a maximum of 16 sessions with a maximum duration of 60 minutes each.

At baseline (T0) the research assistant assesses the baseline measurement of all primary and secondary variables. At 2 weeks (T1) and 4 weeks (T2) into treatment, respectively, participants in the treatment condition are assessed for their scores on paranoia, interaction anxiety, cyber sickness, presence in the virtual world and medication adherence, using self-report questionnaires handed out by the therapist, or interview questions (medication adherence).

At the end of treatment (T3) and at follow-up (T6), the research assistant assesses the primary and secondary measures.

### Randomization

A block of 12 random assignments will be made for each participating mental health center. Each block has six assignments for each condition: VRETp or waiting list. If a center includes more patients, new blocks of 12 random assignments will be made available. The blocks are made using a scientific randomization program (www.randomizer.org) by the independent randomization bureau of Parnassia Psychiatric Institute. Participants and therapists are informed about the randomization by mail and email, respectively.

### Interventions

**Fig. 1:**
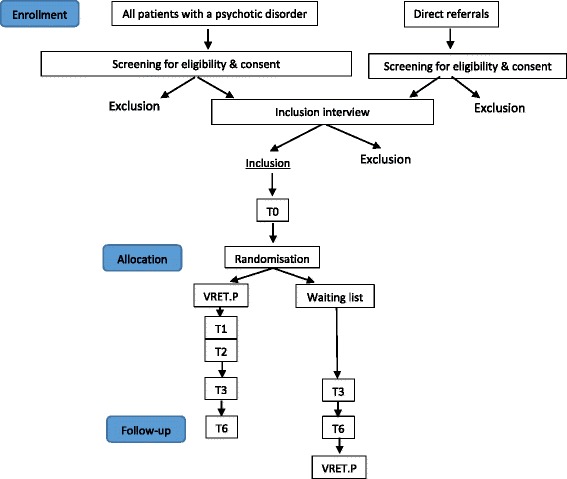
Flow diagram of virtual reality exposure therapy for patients with psychosis (VRETp). A figure showing the flow of patients in the study protocol

Figure 1 is a flow diagram showing study inclusion.

### Treatment as usual (TAU)

Participants in both the VRETp and the waiting list condition receive TAU, consisting of antipsychotic medication, and treatment and/or supportive counseling by therapists, caseworkers or coaches. TAU is considered to be equal in both conditions as a result of the randomization procedure used (see “Randomization”). During the trial participants are not allowed to receive any therapy aimed at improving social participation.

### Virtual reality exposure therapy (VRET)

VRETp treatment has a maximum of 16 treatment sessions of 60 minutes each. This number of sessions is somewhat larger than is usual for CBT in anxiety disorders. Our rationale for this is our expectation that treating paranoia requires more time compared with treating regular anxiety. People suffering from a psychotic disorder often show severity and durance in their social avoidance. Negative symptoms make it difficult to motivate people for therapy and this is a continuing process during treatment. The treatment protocol states that the therapists receive 16 hours of training; all therapy sessions are recorded. A selection of the sessions is rated for treatment fidelity using the Cognitive Therapy Rating Scale (CTRS). The CTRS is a reliable [41] and valid [42] instrument to measure treatment fidelity when following a CBT protocol. Monthly 4-hour group supervision serves to guide the therapists throughout the intervention period.

Existing CBT protocols will be adapted for VRET treatment in one area: exposure in vivo will be replaced by VR exposure. The remainder of the treatment protocol consists of well-known, evidence-based CBT elements, e.g., providing treatment rationale, behavioral experiments, reducing safety behavior, and attention training. Starting with exposure exercises for social situations, which are lowest in the patient’s anxiety hierarchy, the exposure exercises take place during the therapy session using the Virtual Reality system. Participants are not allowed to receive any other form of therapy aimed at improving social participation during the trial. At all times the therapist is in control of the VRETp intervention and is able to immediately change/stop the virtual environment if necessary.

### Early completions

A participant is considered an early completer of treatment when the subjective units of distress on a scale of 0 (no stress at all) to 100 (extremely stressful) in all the virtual situations that are part of the case conceptualization are reduced to zero in two consecutive sessions. This criterion is restrictive and was chosen to prevent therapies being ended prematurely, since no point of reference has been established for treating paranoia using VR exposure.

### Discontinuation

Participation is completely voluntary and participants can withdraw from participation at any time for any reason. Participants who drop out or otherwise deviate from the intervention protocol are requested to continue to participate in the measurements.

### Fidelity checks

Audiotapes are made of all sessions and a selection of them is rated for treatment fidelity. All therapists are supervised by a highly skilled professional (MvdG) to evaluate, guide and approve the case conceptualization. Every 6 weeks, 4-hour group supervision supports the therapist for the duration of the intervention. By means of a questionnaire, for each session the therapist reports on the elements and steps in the treatment protocol. Any deviation from the protocol is reported to the supervisor.

### Unblinding

The study is single-blinded, meaning that research assistants who do the measurements are kept blinded regarding randomization of the participants. Participants are regularly instructed not to tell the research assistants which group they are allocated to; if a research assistant is accidentally unblinded during a measurement, that measurement is stopped. A new appointment is then made with another blinded research assistant.

### Adverse events

The rules and regulations of the Medical Ethics Committee concerning adverse events are followed. All participants are insured in case any harm is caused related to trial participation.

### Analyses

#### Data management

All data are directly coded with a number. Data and personal information of the participants are kept separately and safely stored to ensure privacy. All data entry is double-checked. A data monitor from Parnassia Psychiatric Institute is appointed for the study.

#### Data analysis

Multilevel linear mixed modeling is used to analyze the data according to the intention-to-treat principle. Completer analysis is conducted with analysis of variance (ANCOVA). Moderator and mediator analysis is conducted to assess the effects of moderators and mediators. Cost-effectiveness is conducted with social participation as the outcome.

### Ethics

Ethical approval of the protocol was granted by De Medisch Ethische Toetsingscommissie VU medisch centrum (METC number: NL37356.058.12).

## Discussion

The main goal of the study is to investigate the effect of VRETp on improving social participation in patients with psychosis in real life. We hypothesize that VRETp will increase social participation objectively (time spent in company) and subjectively (experience less anxiety and paranoia during in social situations). Comparison between VRETp treatment and a waiting list condition will provide information about the effectiveness of VR exposure therapy for this population. This should make an important contribution to treatment options for people suffering from psychosis and social isolation. Improving social participation is of great personal value to patients with psychosis who suffer from the consequences of avoiding social situations in daily life.

In addition to social participation, the effect of VRETp on psychological, emotional and social well-being, especially paranoia and interaction anxiety, is explored. Studying these variables may help disentangle the complex phenomena related to social participation in patients with psychosis.

Another aspect of the study is cost-effectiveness. If social participation improves it is expected that patients will become more independent, consume less care and, thereby, diminish costs related to extended health care.

### Trial status

Ongoing.

## Abbreviations

BARS: Brief Adherence Rating Scale
BCSS: Brief Core Schema Scales
BDI-II: Beck Depression Inventory
CBT: Cognitive behavioral therapy
CTRS: Cognitive Therapy Rating Scale
DACOBS: Davos Assessment of Cognitive Biases Scale
ESM: Experience Sampling Measurement
GPTS: Green et al. Paranoid Thought Scales
IC: Informed consent
IPQ: Igroup Presence Questionnaire
ISMI: Internalized Stigma of Mental Illness
MANSA: Manchester Short Assessment of quality of life
MINI: Mini International Neuropsychiatric Interview
RCT: Randomized controlled trial
SBQ: Safety Behavior Questionnaire – persecutory delusions
SIAS: Social Interaction Anxiety Scale
SOFAS: Social and Occupational Functioning Assessment Scale
SSQ: Simulator Sickness Questionnaire
TAU: Treatment as usual
TiC-P: Trimbos/iMTA questionnaire for Costs associated with Psychiatric illness
WAIS: Wechsler Adult Intelligence Scale
WISC: Wechsler Intelligence Scale for Children
VR: Virtual reality
VRET: Virtual reality exposure therapy
VRETp: Virtual reality exposure therapy for patients with psychosis
